# Toward a Demsetzian Knowledge Theory

**DOI:** 10.1007/s13132-022-00901-6

**Published:** 2022-02-11

**Authors:** Vladimir Vladimirovich Maltsev, Andrei Yurievich Yudanov

**Affiliations:** 1 Department of Economic Theory, Financial University Under the Government of the Russian Federation, Moscow, Russia; 2 Research Fellow at Kings College London, Centre for the Study of Governance and Society, London, United Kingdom

**Keywords:** Knowledge encapsulation, Harold Demsetz, Full knowledge, Traditions, Superstitions, Institutions, Division of labor, Outsourcing, B31, D83, D89

## Abstract

The paper attempts to outline a general theory of knowledge in economics based on the work of Harold Demsetz. We identify that “knowledge encapsulation” is a key Demsetzian idea that could unite the otherwise fragmented or narrow research on knowledge in economics. The knowledge encapsulation concept holds that mobilizing cognitive resources and acting under full knowledge is costly. This creates an incentive to compress knowledge into an algorithmic form, which can then be transferred in a cost-efficient manner between a multitude of agents. From this idea of Demsetz, we create a simple theoretical model. To prove its generalizability, we extrapolate it onto a wide range of cases, from traditions and superstitions to institutions and division of knowledge. We conclude that knowledge encapsulation applies to an extensive array of phenomena. However, such encapsulation must also be supplemented by adequate enforcement and mechanisms of coping with ex-post consequences of its use. If our analysis is correct, then the Demsetzian theory could be a strong contender for becoming a fruitful alternative research paradigm on knowledge in economics.

## Introduction

Many scholars hold that today we live in a “knowledge-based” or “knowledge” economy (Foray & Lundvall, 1996; Moisio, 2018), focused on the production and distribution of knowledge (Boettke & Sautet, 2015: 77). The knowledge economy and its related studies focus on the questions of innovation, technological progress, and their influence on the overall economy (Madrak-Grochowska, 2015).

In previous decades, economic theory has attempted to catch up to these developments and move away from the classification of Stigler (1961: 213), where knowledge occupied a “slum dwelling” in the discipline. Numerous scholars tried placing knowledge and information at the center of economic analysis (Hayek, 1937, 1945; Stigler, 1961; Alchian, 1969; Marschak, 1969; Hirshleifer, 1973; Hurwicz, 1973; Koopmans, 1977; Grossman & Stiglitz, 1976, 1980; Sah & Stiglitz, 1985; Thomsen, 1992; Stiglitz, 2000, 2002; Myerson, 2009; Rothbard, 2009; Easterly, 2014; Koppl, 2018). The research on the economics of knowledge since then followed many different paths, from treatment of information as statistical induction to symbolic computation or “measures over partitions of states of the world” (Mirowski & Nik-Khah, 2017: 152). However, the overall project still exhibits a significant research gap up to this day, best summarized by Mirowski and Nik-Khah (2017: 151): “there is still no single orthodox economics of information, even at this late date, although there are trends that can be described, if not completely explained.” Unless such a single theory is created, the current efforts at studying the economics of knowledge are bound to remain disjointed, applicable only to niche fields of inquiry.

Our research goal then lies in moving toward a single theoretical framework for economics of knowledge that is applicable to a large variety of cases. We believe that the concept of *knowledge encapsulation*, devised by Demsetz (1988, 1997), may provide a focal point for such efforts. Demsetz understood knowledge encapsulation as the existence of various algorithms which shape knowledge into an easily digestible form and enable its cost-efficient transfer between economic actors. The logic explicated by Demsetz is vast in scope, as it deals with a problem universally faced by all economic agents, namely, in overcoming the costliness of utilizing full, perfect knowledge. Unfortunately, Demsetz stopped short of any generalizable propositions and mostly focused on the study of firms. As such, it befalls to us to expand the conceptual scope of the Demsetzian knowledge encapsulation, so that it may become a centerpiece in a general theory of knowledge in economics.

Our study has been structured in accordance with our research goal. The second part is a literature review that highlights the lack of a unified theory regarding knowledge in economics and shows how this problem may be overcome through the creation of a Demsetzian alternative. The third part delineates our research methodology. The fourth part outlines our general theoretical model. Fifth part applies this model to a wide range of cases to prove that the Demsetzian approach is broad in its applicability and thus may be considered as generalizable. Sixth part concludes.

## Literature Review

The ambitious task to reframe the central problem of economics as the “utilization of knowledge which is not given to anyone in its totality” began with Hayek (1937, 1945). His insights were picked up by the mainstream economists, but the research has since strayed along different paths.

The created fields of inquiry treated knowledge or information as a public good (Arrow, 1962), human capital (Becker, 1964), a regular commodity (Machlup, 1962), inductive index (Blackwell, 1953), or as an input in computational algorithms (Mirowski, 2002). Other strands of research narrowed themselves to niche, specific areas such as studies of brokers and middlemen (Alchian, 1969), advertising (Rothbard, 2009), expert studies (Easterly, 2014; Koppl, 2018), or institutions, for instance, the market (Rothbard, 2009; Thomsen, 1992). Even Hayek himself sowed confusion in his project, changing his treatment of knowledge “from being merely difficult to retrieve, to being partly inaccessible, to becoming finally so transcendent no one can really know it” (Mirowski & Nik-Khah, 2017: 72).

As a result, these fields of inquiry are bereft of interconnectedness and unifying elements, preventing the formation of one single theory. Nevertheless, some of the studies on knowledge contain common quantifiable elements which may help in creating a general theoretical edifice. These common elements are the costs of knowledge use and learning. Consider this passage in Hayek (1945: 528): “Civilization advances by extending the number of important operations which we can perform without thinking about them.” Furthermore, “We make constant use of formulas, symbols and rules whose meaning we do not understand and through the use of which we avail ourselves of the assistance of knowledge which individually we do not possess.” Foray (2004: 5) continues this insight: “Where knowledge is concerned, the main economic problem is its reproduction (problem of learning), while the reproduction of information poses no real problem (the marginal cost of reproduction is close to nothing).”

More generally, the act of thinking itself is not costless, as the mobilization of an agent’s mental faculties can be expensive. Knowledge transfers are also costly, necessitating a sufficiently prepared recipient. Then, the drive to act “without thinking” is a natural attempt to minimize the costs of cognitive processes, pervasive through all economic action. These insights are elaborated in detail by Demsetz (1988, 1997) but have been frequently ignored by economists.

The research of Demsetz focuses on the costs borne by economic agents in the world of imperfect information. Demsetz (1997: 82) criticized economists who thought that knowledge is “freely converted to maximizing solutions.” Moreover, it is not enough to simply purchase knowledge “in the form of facts, for in many cases the theory that links facts must be mastered if facts are to be put to work” (Demsetz, 1988: 157).

The Demsetzian solution to minimize the knowledge costs lies in the creation of a “low-cost method of communicating between specialists and the large number of persons who either are non-specialists or who are specialists in other fields” (Demsetz, 1988: 157). These methods are then realized through knowledge encapsulation, where full knowledge is converted into a less costly and easily transferrable form. The Demsetzian ideas are affirmed by Mirowski and Nik-Khah (2017: 107), who note that real-life transactions that deal with knowledge always involve sales of derivative objects, to which full knowledge is condensed due to its costliness. Thomsen (1992: 41–45), also claims that the “economy of knowledge” creates “knowledge surrogates,” which compress knowledge to a point where little of it is demanded from agents to act.

This Demsetzian logic is our impetus toward the formation of a unified theory of knowledge in economic theory. Let us proceed by discussing its relevant methodology.

## Research Methodology

Our methodology falls squarely into the “theory adaptation” vein of conceptual research design (see, for instance, Jakkola, 2020). We have already designated the theories of Hayek on knowledge and their neoclassical iterations as a starting point and justified the need for a shift in their focus. The Demsetzian theory of encapsulation was then suggested as an alternate focal point in economics of knowledge. The use of encapsulation at the center of knowledge economics will also switch the theoretical focus from knowledge creation to the process of moving from full to encapsulated knowledge.

To supplement our efforts, we want to carefully delineate the concept of knowledge encapsulation through creating a simple model. Our model will outline the key variables associated with the phenomenon and explain the relations between them. We will break down the components of full knowledge and offer a demand model to explain what incentivizes agents to move from full to encapsulated knowledge. This clarification will offer greater tractability to the focal point of our paper and unveil its relevant constraints.

According to the theory adaptation paradigm, we must also show why the Demsetzian theory is the best available option for the creation of a general theory of knowledge in economics. For that, we seek to apply it to a large variety of cases to affirm its scope. Our reasoning is simple: if a theory can apply to a broad number of scenarios, then its overall generalizability may be categorized as high. Case studies are overall characterized by Yin (2013) as providing research with analytical depth, conceptual validity, and deeper understanding of underlying processes in the considered phenomenon. Matching the proposed theory with cases also enables more sophisticated analytical techniques to be employed in future studies (Ridder, 2017). Our cases will be based on real-world historical examples and scenarios, tackling algorithms of encapsulation from instructions and directions to the more exotic cases of traditions, superstitions, institutions, and artificial intelligence.

## A Simple Theoretical Model

Imagine an economic agent that needs to act autonomously. In such a situation, the agent requires full knowledge. We define full knowledge as “knowledge necessary for autonomous decision-making” and introduce six elements that comprise it:

1. Socially acceptable or objectively true view of the world,
2. Factual data/information relevant to the problem,
3. An arsenal of relevant skills, methods, and technologies,
4. An action algorithm,
5. Enforcement of knowledge,
6. A mechanism for coping with ex-post consequences of knowledge use.

Foray and Lundvall (1996: 115–116) describe the first three elements as different “knows.” Element number one is a “know-why,” which encompasses objective or socially acceptable principles and laws. Element number two is a “know-what” — bits and pieces of information, best available to experts in their relative fields. Element number three is a “know-how” — the capability to do something.

Element four represents some algorithm, a step-by-step fulfillment of a knowledge-based action. From a practical perspective, this is the most important element.

Elements five and six are usually ignored under full knowledge, as an individual acting on full knowledge fully realizes its consequences. Thus, the agent has no need to devise any mechanisms for coping with its ex-post use. Furthermore, there is no need to enforce the use of this knowledge as the decision to act upon it is perfectly aligned with the end goals set before the agent (Demsetz, 1997: 9).

The costliness of full knowledge is concentrated in elements one, two, and three. These costs are further augmented by how expensive it is to process this knowledge through the agent’s cognitive resources. We can thus postulate that the demand for full knowledge depends not on one, but two prices: the price of knowledge set by its producer and the price of cognitive resource mobilization by the consumer:

1 $${D}_{{f}\_{kn}}=f({P}_{{kntr}}, {P}_{{cogn}\_{res}})$$

where ${D}_{{f}\_{kn}}$ is the demand for full knowledge, ${P}_{{kntr}}$ is the price of knowledge transfer by the producer, and ${P}_{{cogn}\_{res}}$ is the price of mobilizing the cognitive resource by the knowledge consumer. To present this in the simplest linear form:

2 $${D}_{{f}\_{kn}}=a-b({P}_{{kntr}}+ {P}_{{cogn}\_{res}})$$

where *a* and *b* are coefficients. As a result, the demand for full knowledge becomes zero or negative, if:

3 $${P}_{{cogn}\_{res}}\ge \frac{a}{b}-{P}_{{kntr}}$$

Such a situation might be encountered when rational reasoning is utilized to persuade skeptically minded individuals. In this case, the costs of mobilizing the cognitive resources will be very high, which leads to negative demand even under significant enforcement potential^1^. The inequality (Eq. 3) is also suitable for describing situations where the demand for full knowledge may be negative, even if ${P}_{{kntr}}$ = 0. If the value of ${P}_{{cogn}\_{res}}$ is too high, then the mobilization of cognitive resource is prohibitively expensive^2^.

An opportunity to reduce the costs of full knowledge incentivizes agents to move to incomplete knowledge. The Demsetzian solution to the problem of full knowledge costs focuses on element four — the encapsulation of knowledge into algorithms. An algorithm itself is a necessary full knowledge element for our autonomous agent, as it embodies a practical application of condensed insights obtained from the first three elements. However, once the algorithm has been established, it can be transferred to other economic agents without loss of key features which enable action. The algorithm consumers can simply follow it step by step, without incurring the costs of cognitive resource mobilization from elements one, two and three.

Then, when a consumer is involved in a transfer of encapsulated knowledge, the price of cognitive resource mobilization equals to zero, or

4 $${D}_{{e}\_{kn}}=a-b{P}_{{kntr}}$$

where ${D}_{{e}\_{kn}}$ is the demand for encapsulated knowledge. Correspondingly, the total change in the demand by moving from full knowledge to encapsulated knowledge is

5 $${D}_{{e}\_{kn}}-{D}_{{f}\_{kn}}=b{P}_{{cogn}\_{res}}$$

Let us also consider that knowledge production costs are largely fixed. The cost of creating an algorithm is certainly dependent on the knowledge contents that need to be compressed, but the transfer volume matters little due to almost zero marginal costs of replication. As such, the average costs of knowledge encapsulation can decrease proportionally to the demand growth. The encapsulation of knowledge thus also increases the efficiency of producing said knowledge:

6 $${AC}_{{e}\_{kn}}={AC}_{{f}\_{kn}}\frac{{D}_{{f}\_{kn}}}{{D}_{{e}\_{kn}}}$$

However, the use of algorithms involves a trade-off. Reliance on algorithms can increase the costs of full knowledge elements five and six, which play a more significant role under encapsulated knowledge.

Having created our simple theoretical model, let us now test its scope and general applicability.

## Case Studies on Knowledge Encapsulation

We created the following list of widely applicable knowledge encapsulation methods:

(A) Direction,
(B) Instruction,
(C) Tradition (including superstition),
(D) Institutions as information catalysts,
(E) Division of labor,
(F) Knowledge outsourcing.

Demsetz (1988: 157) himself elaborated on knowledge encapsulation by providing examples of directions, where “Direction substitutes for education” or user instructions and manuals bundled with goods. Directions, issued within a hierarchy, allow those who do not possess enough specialized knowledge to be directed by specialists^3^. A firm’s manager can issue directions to the employees instead of engaging in costly explanations. This allows a firm to greatly economize on element three of full knowledge. Directions do not require additional enforcement costs, as relevant mechanisms already exist within the established hierarchy. At the same time, the creation of the hierarchy itself might be costly.

Alternatively, instructions may be employed. Instructions allow greater discretion about their timing and application. The use of instructions economizes on full knowledge element three by substituting a qualified specialist with a worker of lesser qualifications. Training a qualified specialist is costly, but a non-qualified worker can simply follow instructions to the letter. The success of artificial intelligence in healthcare also shows a situation in which an instruction can exist without any knowledge of other agents whatsoever. A significant degree of data formalization via laboratory analysis, MRI scans, cardiograms, and ex-post evaluation of medical decision-making through anatomical pathology creates favorable conditions for AI deep learning. Currently, some results of AI diagnosis are superior to those made by medical professionals (Abràmoff et al., 2018). The use of instructions, in this case, is straightforward and is reduced to inputting data into a program for AI assessment. The result of such a process is likely to be superior and obtained at a lesser cost.

Some instructions require additional enforcement costs, especially if the incentives of the economic agent do not perfectly align with the intended use of the instruction. Many agents do not fully adhere to technology instructions and safety precautions, as strict adherence slows down the process of action. However, if the fulfillment of safety measures is crucial for the instruction user’s life, then the instruction must be supplemented through controlling agencies. These organizations increase the costs of full knowledge element five, associated with enforcement. This is precisely what happens in the aviation industry, and it is overseen through such organizations as the International Civil Aviation Organization. If enforcement of knowledge in the industry is costly, it may result in higherex-post costs through air crashes, the majority of which are caused by a human factor (Shappell et al., 2007). Then, while significantly reducing the costs of full knowledge elements one, two, and three and broadening the scope of users, the instruction can cause a corresponding increase in costs for elements five and/or six.

Traditions encompass another broad area of knowledge encapsulation. Traditions function most effectively when the costs of full knowledge elements two and three are high. Knight (1947: 90) states that “Primitive society was wise in its conservatism, for it knew at least that the group had previously lived somehow, both as individuals and as a group.” Taleb (2012) also devotes much attention to traditions. He defines traditions as “tacit, unexplainable rules of thumb transmitted through generations” (Taleb, 2012: 215). Taleb claims that traditions are important as they represent vital knowledge, crystallized in entrenched societal practices.

Traditions create a cost-effective channel of enforcement through influencing societal opinion. It follows a procedure of “do it this way, because that is how our ancestors used to do it.” Individuals who go against the rules may risk being ostracized by the community. This punishment mechanism ensures that encapsulated knowledge is enforced. High social homogeneity can further ensure the enforcement of traditions. Consider the case of Japan in the light of the recent COVID-19 pandemic. The established culture of wearing masks and the various traditions such as keeping self-organized queues in the subway allowed for a more efficient way to enforce pandemic-related knowledge.

The main counterpoint to employing traditions as knowledge-encapsulating vehicles is whether such behavioral algorithms are welfare-enhancing. Here we can advance an evolutionary argument contained in the works of Stigler^4^ (1992: 459), namely that “all durable social institutions … must be efficient.” Societies that follow inefficient traditions do not survive. On the other hand, traditions that are entrenched in prospering societies, are, perhaps, efficient.

Let us consider a seemingly worse case than traditions — superstitions. In the last decade, there has been an influx of research that attempts to prove the rationality behind many superstitions. Leeson (2012a: 185) defines superstitions as “objectively false beliefs that individuals hold.” The main impetus of his articles is that “many objectively false beliefs *improve* social cooperation and productivity” (Leeson, 2012a, b, 2014a, b). A strong case can be made to treat superstitions as knowledge encapsulation vehicles, which can significantly reduce the costs of full knowledge associated with elements two and three.

Let us consider the case of pork-eating prohibition in the Islamic faith^5^. The Quran (6:145) refers to the consumption of pig flesh as religiously “impure.” It is a known scientific fact that the consumption of improperly inspected pork meat can lead to trichinosis — a disease spread through the Trichinella Spiralis helminth (Djurkovic-Djakovic et al., 2013). The spread of the parasite could be further magnified by the quicker decay of meat in the hot and arid climate of the Arabian Peninsula. Without the advances in modern medicine, trichinosis outbreaks could have been extremely deadly in the past. Consider the mobilization of the cognitive resource needed to explain the potential dangers of eating pork to a layman during this historical period. This would require the explanation of what parasites are, why they are contained in pork meat, how they infect individuals, and how severe this infection can manifest. Contrast this lengthy explanation to a cost-effective algorithm of “God forbids eating pork. And we must obey God.” Steadfast belief in superstitions can enforce the use of these algorithms. In our case, this can be done via the threat of incurring the wrath of God and not entering Paradise — an extremely high cost for the true adherents of the faith^6^. Thus, consistently, the Demsetzian logic of knowledge encapsulation can be productively applied to even such an extreme case.

Institutions exhibit some similarities with traditions as knowledge encapsulation algorithms. A classic example exhibited by Hayek is the role markets play in encapsulation of an immense volume of knowledge^7^ on supply and demand dispersed amidst a vast number of economic agents. The market as an institution encapsulates dispersed knowledge and ensures that it reaches every market participant in a barebones form. However, this form — a price, is nonetheless sufficient for making a practical decision. This affects the full knowledge element two. In his work “The Use of Knowledge in Society,” Hayek (1945: 526–527) illustrates this phenomenon with a hypothetical example of a tin market, which faces an increase in scarcity of this material. However, for the economic agents operating on this market “There is no need for the great majority of them even to know where the more urgent need [for tin] has arisen.” All that they need to know is that “some of the tin they used to consume is now more profitably employed elsewhere, and that in consequence they must economize tin.”

It can be argued that knowledge encapsulation makes the very existence of the market economy possible. Without it, the only other alternative would be the establishment of a Central Planning Bureau, where all economic problems would be resolved via a central agent in possession of full information. However, the costs of cognitive resource mobilization on a nation-wide scale and collection of an immense volume of data are very significant. In the case of the Soviet Union, this led not only to deficits and the overall decrease in an assortment of goods available, but also to the functioning of extralegal market activity and “blat” through which individuals strived to reduce at least some of the knowledge costs through highly distorted quasi-market prices.

It is quite notable that in almost seventy years that have passed since the publishing of the “Use of Knowledge in Society,” there have been very few other scholarly examples of the role that institutions play as vehicles of encapsulation (see for instance, Stigler, 1961; Alchian, 1969; Rothbard, 2009). Perhaps a direct transformation of full knowledge to incomplete is a function rarely performed by institutions.

Additionally, institutions play a significant role in limiting the risks associated with the use of encapsulated knowledge. Consumer organizations or consumer rights laws guarantee that the consumer will not take unnecessary risks using incomplete knowledge. When using a smartphone, a user has the confidence that he will not suffer death from the explosion of a battery pack inside the device. Such assurance is necessary, as without additional regulatory institutions, any user operating in a situation of incomplete knowledge may not know the potential consequences of his own actions. Risk minimization as seen through the encapsulation process provides examples like patent rights, employment laws and culturally institutionalized lifetime employment practices in Japan. This process can also be seen in areas with high levels of trust and social homogeneity. In other words, good institutions can radically minimize the ex-post costs of using incomplete knowledge.

The division of labor also falls under the umbrella of knowledge encapsulation. It is hard to dispute that economic actors cooperate under the division of labor. Division of labor leads to specialization, where individuals encapsulate knowledge into material objects. These material objects then allow their users to tap into the encapsulated knowledge they contain without the need to focus on element one of our full knowledge definition. A child or a completely uneducated individual can use a smartphone without understanding the fundamental principles of how the gadget functions. The encapsulation of knowledge does not materialize through the physical instructions that the smartphone is supplied with, and which are frequently not even read. The instruction and the action algorithm of using a smartphone are embedded into its interface, software, and hardware. As a result, smartphone users enjoy cost reductions of almost all full knowledge elements.

The encapsulation of knowledge is especially potent if the division of labor follows a modular pattern. If full knowledge is comprised of various blocks that exist independently, then all the necessary information must be transferrable between these blocks in a cost-efficient form. To provide an example from Demsetz (1988: 158), “The economical use of industrial chemicals by steel firms does not generally require knowledge of how these chemicals are produced; similarly, the use of steel by industrial chemical firms does not require the transfer of knowledge of how the steel is produced.” Knowledge encapsulation must adhere to a specific set of characteristics set by a given module. For instance, a steel firm can demand a particular acid with sufficient potency, necessary to remove impurities from the steel.

Perhaps due to the widely spread modular structure of knowledge, the division of labor plays a significant role in economic development. Such treatment allows us to take a different perspective on the famous example of Adam Smith’s pin factory. As Smith (2007: 3) describes it, the production of the factory was not automated, and as such, gains from production must have accrued due to the workers economizing on the use of knowledge by transferring it between each other in various specialized blocks, shaped by “distinct hands.”

Another possible way of using the division of labor as an encapsulation method is through knowledge outsourcing, which in our context implies the transfer of decision-making. In this case, an economic agent does not seek to receive a block of knowledge. Instead, the economic agent must search for a person or a device such as artificial intelligence, to whom he will relegate the solution of knowledge-related tasks. The agent then focuses only on his desired ends and dealing with the ex-post results of the process.

In the era of GPS navigators, the need for car drivers to know the cities they live in has decreased drastically. However, before such devices existed, individuals already outsourced topographical knowledge tasks to postillions, as written in Voltaire (1901) “Jeannot and Colin.” In this small story, the father asks his son’s governor about what sciences his child should be versed in, bringing up geography. To that, the governor replies: “Of what use will that be? When the marquis goes to his estate, won’t the postillion know the roads?”.

Let us try to sum up our findings in the form of a table. The rows of the table represent various encapsulation methods, apart from the full knowledge row, which is considered as a benchmark of sorts. The columns represent the elements of full knowledge, as provided in our definition. The cells either contain the word “high” or “low,” to indicate the level of costs we can expect from employing a particular encapsulation method concerning a given element of full knowledge. The last column of the table contains an elaboration on where a particular knowledge method can be used. The aggregated results are presented in Table 1.

**Table 1:** The knowledge costs under various methods of encapsulation

	Correct view of the world	Factual data	Skills	Algorithm	Enforcement	Ex post consequences	The sphere of use
Full knowledge	High	High	High	High	Low	Low	Autonomous action by specialists
Instruction	Low	Low	Low	Low	High	High	Indeterminate number of users
Direction	Low	Low	Low	Low	Low	High	Hierarchical structure
Tradition	High	Low	Low	Low	Low	High	Society as a whole
Institutions	Low	Low	Low	Low	High	Low	Society as a whole
Division of labor	Low	Low	Low	Low	Low	High	Market participants
Knowledge outsourcing	Low	Low	Low	Low	Low	High	Secondary decision-making

Let us note that economic agents must also make a choice between alternate methods of knowledge encapsulation, for instance, between using directions or the division of labor. One of the few examples by Demsetz (1988: 147–148) touched upon an optimal level of vertical integration and its corresponding management dilemma of “do it yourself” versus “buy it from someone else.” The process of adding more and more stages to the value chain ends when the costs of agent relations within a firm begin to exceed the costs of external contracting. At some point, it becomes more cost-efficient to halt the vertical integration and simply purchase similar goods that contain relevant encapsulated knowledge from a different firm.

Another choice can be made between instructions versus institutions. For instance, it is frequently discussed that firms do not provide sufficient safety for consumers, which results in fires in shopping malls, sinking of cruise ships and so forth. The cause of such incidents can lie in the conscious breaking of safety instructions by the businessmen. To lower their costs, sometimes businessmen can use flammable materials or overload the ships with passengers. Traditionally, safety instructions can be enforced through public control agencies. However, these agencies do not always yield satisfactory results due to negligence or corruption. An alternate way to resolve this is via the institution of mandatory insurance. An insurer is no less fallible than a government bureaucrat. However, the insurer has a “skin in the game” in that he bears the full costs of insuring non-compliant businesses. Such an institution can then reduce the enforcement costs of instruction use.

## Conclusion

In this paper, we created an outline of a general theory of knowledge in economics through the Demsetzian knowledge encapsulation framework. The knowledge encapsulation concept is broad enough to cover a wide variety of cases under its aegis, and we attempted to show such by utilizing in a plethora of cases. With this, we may claim to have at least partly uncovered the knowledge encapsulation’s potential in the formulation of a general economic theory of knowledge.

Our line of research can continue in two directions. Firstly, our list of knowledge encapsulation methods may be incomplete. It may be necessary to extend the Demsetzian framework to continue proving its generalizability. One of the knowledge encapsulation algorithms worth studying is trust. Trust dramatically decreases the costs of full knowledge, as an economic agent simply needs to follow the lead of someone he trusts.

Secondly, knowledge encapsulation algorithms do not exist in a vacuum and can potentially affect other areas of the economy. For example, the overreliance on outsourcing can create the problems of monopoly and power in a similar vein that Koppl (2018) describes in his work on experts. Uncovering more “unintended” effects of knowledge encapsulation is then crucial to allow for a fuller cost–benefit analysis of their implementation.

The last, but the most crucial aspect of our problem is related to the different scopes of full and encapsulated knowledge. The latter is more limited than the former, but it is precisely this limitation which enables knowledge to be integrated into the market economy. Encapsulated knowledge is in such high demand because its algorithms work in most cases. However, sometimes there may exist unforeseen situations that lead to algorithms producing undesirable results. In this case, an economic agent who utilizes encapsulated knowledge must cooperate with the agents in possession of full knowledge. The problem becomes even more pronounced if we consider specific attributes of knowledge as a factor of production, from its non-rivalrous consumption to its ease of copying. Could continuous interactions between full knowledge and encapsulated knowledge ameliorate such a problem, and if so, what form will they take? This warrants additional studies.

## Data Availability

All data and materials support their published claims and comply with field standards.

## Footnotes

1 For instance, the Indian government currently struggles in making its population comply with modern sanitary norms, which oppose the long-standing traditions of burial rituals or bathing in sacred rivers. The cognitive resource mobilization for the traditionally minded populace is so expensive that the demand for the new knowledge is non-existent even during the COVID-19 pandemic.

2 Such a situation might occur under state subsidized education, where cognitive resource mobilization is the key obstacle in creating the knowledge demand for poorly performing students.

3 We can also go beyond the Demsetzian case of knowledge cost minimization between specialists and non-specialists. For instance, knowledge encapsulation is widely used in the medical profession, enabling faster communication between specialists. Instead of engaging in a detailed pathophysiological explanation, professionals exchange keywords or concepts with each other, rendering meaning through those keywords. According to Schmidt and Rikers (2007: 1135), “the concept of sepsis is sufficient to explain all relevant signs and symptoms; it encapsulates the … detailed pathophysiological explanation”.

4 However, Stigler is not the first scholar to be credited with such line of reasoning. In fact, it predates Stigler by many centuries in the works of Pope (2018) and his “Essay on Man”, where he writes that “Whatever is, is right.” Hegel (1991) can also be credited here, with his phrase “what is reasonable is real; that which is real is reasonable”.

5 We must make a disclaimer here to prevent any offenses taken by Islamic or other religious readers: by treating some aspects of the Islamic faith as superstitious, we do not intend to dismiss the entirety of Islam, nor do we wish to claim that all religion should be abolished or deemed useless. As the following passages show, our stance on it is quite the contrary.

6 An alternative to a religious approach could have been the creation of some specialized institution, for instance, a high tax on the ownership of pigs. The Ottoman Empire had a similar tax towards representatives of non-Islamic religions. Such individuals could still practice their faith if they paid a special tax called jizya. Historical documents confirm that Ottoman courts did not persecute Christians for the consumption of wine, as long as they paid jizya (Skvoznikov, 2015: 9). The tax would be lifted after conversion to Islam and subsequent adherence to its customs, including abstinence from wine and pork consumption.

7 It would perhaps be more contemporary to instead refer to this term as “big data”.
